# CRISPR/Cas-Based Molecular Diagnostics for Intestinal Parasites: From Proof-of-Concept to Point-of-Care Testing

**DOI:** 10.3390/diagnostics16203424

**Published:** 2026-10-21

**Authors:** Isra M. Alsaady

**Affiliations:** 1 Department of Medical Laboratory Sciences, Faculty of Applied Medical Sciences, King Abdulaziz University, Jeddah 21589, Saudi Arabia; ialsadi@kau.edu.sa; 2 Special Infectious Agents Unit, King Fahad Medical Research Centre, Jeddah 21589, Saudi Arabia

**Keywords:** CRISPR/Cas, intestinal parasite, POC, molecular diagnosis

## Abstract

Intestinal parasitic infections remain a significant global public health concern, particularly in low- and middle-income countries, where timely and accurate diagnosis is essential for effective disease management and control. Although conventional diagnostic methods, including microscopy, antigen detection, and polymerase chain reaction (PCR), are widely used, each possesses inherent limitations related to sensitivity, turnaround time, laboratory infrastructure, or point-of-care applicability. Recent advances in clustered regularly interspaced short palindromic repeat (CRISPR)/CRISPR-associated (Cas) systems have led to the development of highly sensitive and rapid molecular diagnostic platforms suitable for decentralized testing. This review summarizes current applications of CRISPR/Cas-based diagnostics for intestinal and foodborne parasitic infections, with particular emphasis on assays developed for *Giardia duodenalis*, *Cryptosporidium parvum*, *Clonorchis sinensis*, *Fasciola hepatica*, and *Schistosoma* spp. Published studies have predominantly employed Cas12a, with additional applications of Cas12b and Cas13a and, more recently, a type I-A Cascade–Cas3 system, generally coupled with isothermal amplification methods such as recombinase polymerase amplification (RPA) or recombinase-aided amplification (RAA), enabling highly sensitive detection within 60–90 min using fluorescence or lateral-flow strip readouts. The available evidence indicates high analytical sensitivity and specificity, with several assays demonstrating substantial agreement with conventional molecular methods and potential for point-of-care development. However, current CRISPR-based assays remain limited by dependence on nucleic acid extraction, relatively small clinical validation cohorts, lack of standardized protocols, and the persistence of false-negative results associated with low parasite burdens and pre-analytical variables. Emerging strategies, including simplified or extraction-free sample preparation, one-pot sample-to-answer platforms, and detection of parasite-derived cell-free DNA, may further enhance the clinical utility of CRISPR diagnostics. Overall, CRISPR/Cas-based technologies represent a promising next-generation approach for intestinal parasite diagnosis, although large-scale clinical validation and workflow optimization are still required before widespread implementation in routine clinical and field settings.

## 1. Introduction

The effectiveness of molecular diagnostic techniques for the detection of intestinal parasites is influenced by several key factors, most notably parasite abundance, DNA extraction efficiency, and the sensitivity of the detection method. Previous research has demonstrated that low parasite loads can significantly reduce detection sensitivity, even when highly advanced molecular tools are employed. In particular, the DNA extraction step plays a critical role, as inefficient lysis or purification can lead to poor DNA recovery and subsequent false-negative results. This is especially relevant for PCR-based detection of eukaryotic enteric parasites, which relies heavily on the extraction of high-quality DNA [1]. However, DNA extraction from faecal samples presents unique challenges, as such samples often contain a wide range of inhibitory substances that may co-extract and interfere with downstream PCR amplification [2,3]. Additional technical limitations, including incomplete cell lysis and DNA fragmentation, can further compromise the efficiency and reliability of molecular detection [4,5]. Table 1 summarizes the principal diagnostic techniques used for intestinal parasite detection.

As summarized in Table 1, available diagnostic methods for intestinal parasites involve important trade-offs between analytical sensitivity, infrastructure requirements, turnaround time, and point-of-care applicability. Despite advances in molecular diagnostics, routine diagnosis continues to rely heavily on conventional microscopy, while molecular methods remain limited by equipment requirements and the challenges associated with nucleic acid extraction from stool specimens. These challenges are particularly relevant to intestinal parasites because of variable and often low parasite burdens and the complex stool matrix. CRISPR/Cas-based diagnostics may therefore provide an opportunity to develop more standardized, sensitive, and accessible molecular approaches specifically suited to intestinal parasite detection.

Consequently, considerable attention has shifted toward integrating isothermal amplification technologies with CRISPR/Cas systems to develop rapid, portable, and highly sensitive diagnostic platforms.

## 2. Literature Search Strategy

A narrative literature search was conducted using PubMed Central (PMC), ScienceDirect, and Google Scholar to identify published studies investigating CRISPR/Cas-based diagnostics for intestinal parasites. The search employed combinations of keywords, including “CRISPR,” “Cas12,” “Cas13,” “Cas3,” “intestinal parasites,” “parasitic infections,” “diagnosis,” and “point-of-care testing.” The final literature search was conducted on 1 September 2026.

Studies investigating CRISPR-based detection of intestinal parasites were considered for inclusion, including those involving human clinical specimens, animal samples, and foodborne parasites of medical importance. Studies focusing exclusively on non-intestinal parasites or parasites without relevance to human intestinal infections were excluded to maintain the scope of the review.

Selected studies were examined for their diagnostic methodology, analytical sensitivity, clinical validation, and potential applicability to point-of-care testing.

## 3. CRISPR/Cas Diagnostic Principles:

The integration of CRISPR/Cas systems with isothermal amplification technologies has significantly advanced the development of rapid and sensitive molecular diagnostic assays. Among these approaches, recombinase polymerase amplification (RPA) combined with CRISPR/Cas detection has emerged as a promising strategy for point-of-care testing (POCT). RPA amplifies nucleic acids under isothermal conditions (37–42 °C) without requiring thermal cycling [12]. Unlike conventional PCR, which requires repeated temperature changes and sophisticated thermocyclers, RPA relies on recombinase enzymes, single-stranded DNA-binding proteins, and strand-displacing polymerases to achieve rapid amplification, typically within 15–30 min. When combined with CRISPR/Cas systems, RPA provides rapid target amplification, while sequence-specific CRISPR-mediated recognition enables subsequent detection through fluorescence or lateral-flow readouts. [5].

Among isothermal amplification technologies, recombinase polymerase amplification (RPA) has emerged as a promising strategy for point-of-care testing (POCT). RPA amplifies nucleic acids under isothermal conditions, typically at 37–42 °C, without requiring thermal cycling. Unlike conventional PCR, RPA relies on recombinase enzymes, single-stranded DNA-binding proteins, and strand-displacing polymerases to achieve rapid amplification, generally within 15–30 min. When combined with CRISPR/Cas systems, RPA provides rapid target amplification, while sequence-specific CRISPR-mediated recognition enables subsequent detection through fluorescence or lateral-flow readouts. Its relatively low operating temperature also permits the use of simple heating devices, supporting the development of decentralized diagnostic workflows [5,13,14].

Compared with loop-mediated isothermal amplification (LAMP), RPA operates at a lower temperature (approximately 37–42 °C versus 60–65 °C) and uses a simpler primer design, typically requiring two primers rather than the four to six primers commonly used in LAMP. These characteristics facilitate its integration with CRISPR/Cas systems, particularly Cas12a, which operates efficiently at a similar temperature. LAMP, however, provides robust amplification and can also be successfully coupled with CRISPR detection, although its higher reaction temperature and more complex primer design may complicate one-pot integration. Accordingly, RPA has been more frequently adopted in the intestinal parasite CRISPR assays reviewed here, particularly where simplified workflows and lower equipment requirements are prioritized [15].

A typical CRISPR/Cas12a diagnostic assay consists of a Cas12a protein, a target-specific CRISPR RNA (crRNA), and a labeled single-stranded DNA reporter, usually combined with an upstream amplification reaction. Following recognition of a target DNA sequence adjacent to a compatible protospacer adjacent motif (PAM), the Cas12a–crRNA complex undergoes conformational activation and indiscriminately cleaves surrounding single-stranded DNA reporter molecules. This collateral nuclease activity transforms molecular recognition into an easily detectable signal, which can be measured using fluorescence or lateral-flow strips [16,17]. Unlike conventional PCR, which generally requires real-time fluorescence detection systems, CRISPR/Cas12a assays can generate visible results using portable fluorescence readers or lateral-flow devices that are interpreted by the naked eye. However, their analytical sensitivity and specificity depend on target selection, guide RNA design, amplification performance, and sample quality.

Building on these characteristics, the DNA Endonuclease-Targeted CRISPR Trans Reporter (DETECTR) platform integrates RPA-mediated target amplification with Cas12a-based detection, enabling rapid, highly sensitive nucleic acid detection under isothermal conditions [18,19]. The complete workflow can typically be completed within 30–60 min and requires only minimal laboratory equipment, potentially simplifying its implementation in decentralized settings These features make CRISPR/Cas12a particularly attractive for point-of-care applications in resource-limited settings, field surveillance, and outbreak investigations, where conventional molecular diagnostics are often unavailable. Moreover, compatibility with one-pot reaction formats, extraction-free sample preparation, smartphone-assisted image analysis, and lateral-flow strip readouts has further accelerated the development of fully portable sample-to-answer diagnostic platforms [20,21,22,23,24]. As summarized in Table 2, these technological advances have enabled the application of CRISPR/Cas12- and Cas13-based assays to several intestinal and foodborne parasites, with promising analytical performance and potential for point-of-care implementation.

## 4. CRISPR/Cas Applications in Intestinal Parasite Diagnosis

The earliest CRISPR-based diagnostic platform for intestinal parasites was reported by Yu et al. [11] who developed the Recombinase Polymerase Amplification–CRISPR/Cas12a (ReCTC) assay for the detection of *Cryptosporidium parvum* IId-subtype-family. The assay targeted the highly polymorphic *gp60* gene and represented one of the first demonstrations that CRISPR technology could be successfully adapted for protozoan parasite diagnosis. Following RPA, Cas12a-mediated collateral cleavage was used to generate fluorescence and lateral-flow strip signals, allowing rapid visual detection without sophisticated laboratory equipment. The assay achieved an analytical sensitivity of one target DNA copy or one oocyst/mL by fluorescence and demonstrated no cross-reactivity with other *Cryptosporidium* subtype families or common intestinal protozoa. Importantly, clinical validation using calf and human faecal samples showed complete agreement with nested PCR and sequencing. One of the most innovative aspects of this study was the introduction of a simplified crude DNA extraction protocol using N-lauroylsarcosine sodium salt (LSS) combined with a homemade DNA-binding column, reducing dependence on commercial extraction kits. This represented an important step toward field-deployable CRISPR diagnostics and addressed one of the principal barriers limiting molecular diagnosis in resource-limited settings.

Following the successful application of Cas12a for protozoan parasites, Huang et al. [25] extended CRISPR diagnostics to the foodborne trematode *Clonorchis sinensis*. Their Cas12a-RPA assay targeted the ITS1 region and was evaluated using both human faecal specimens and infected fish samples. The assay achieved an impressive analytical sensitivity of one DNA copy/μL and demonstrated complete concordance with nested PCR. The dual fluorescence and lateral-flow strip detection formats further highlighted the versatility of Cas12a-based diagnostics for both laboratory and field applications, illustrating the feasibility of integrating CRISPR into surveillance programs for zoonotic parasitic diseases.

During the same year, MacGregor et al. [26] introduced a different CRISPR platform by applying the Cas13a-based SHERLOCK system for the diagnosis of *Schistosoma japonicum* and *S. mansoni*. Unlike Cas12a, which targets DNA, Cas13a recognizes RNA molecules following amplification, thereby expanding the diversity of CRISPR effectors available for parasitic diagnostics. Their assay achieved extremely high analytical sensitivity, detecting as little as 0.001 pg/μL for *S. mansoni*, while maintaining excellent specificity. In addition, the study demonstrated the applicability of the assay to both stool and serum samples, suggesting the potential utility of blood-based detection of circulating parasite-derived nucleic acids. Nevertheless, compared with Cas12a-based platforms, the SHERLOCK workflow remained relatively complex, requiring additional processing steps that may limit immediate translation to routine point-of-care testing.

The year 2024 marked a significant expansion in CRISPR-based diagnostics for intestinal parasites, with multiple studies demonstrating improved assay performance and broader clinical validation. Yang et al. [27] developed a Cas12a-RPA assay targeting the mitochondrial COX1 gene of *Fasciola hepatica*. Validation using both sheep and human faecal samples demonstrated excellent diagnostic agreement with conventional molecular methods, an analytical sensitivity of 0.122 copies/μL, and no cross-reactivity with related parasites. These findings confirmed the high specificity and clinical applicability of Cas12a-based diagnostics for trematode infections.

Building upon this work, Li et al. [28] evaluated the use of Cas12b coupled with recombinase-aided amplification (RAA) for the detection of *F. hepatica*. Although the analytical sensitivity (2.6 copies/μL) was slightly lower than that achieved by Cas12a, the study demonstrated that alternative Cas effectors could also be successfully incorporated into parasite diagnostics while maintaining excellent diagnostic agreement with PCR-based methods. This work broadened the range of CRISPR enzymes available for molecular diagnosis and highlighted the flexibility of CRISPR platform design.

Two independent studies further expanded CRISPR diagnostics to *Giardia duodenalis* [29,30]. Zhao et al. [30] developed a Cas12a-RPA assay that successfully detected *G. duodenalis* in both human and cattle faecal samples with a limit of detection of 10^−1^ copies/μL and no cross-reactivity with nine common enteric pathogens. Clinical evaluation showed complete agreement with nested PCR, supporting the feasibility of CRISPR-based diagnosis in both veterinary and human medicine.

More recently, Wang et al. [29] introduced the REPORT platform specifically targeting *G. duodenalis* Assemblages A and B using the *tpi* gene. This assay represented one of the most clinically advanced CRISPR applications currently available for intestinal parasites. In addition to achieving excellent analytical sensitivity, it differentiated clinically important *Giardia* assemblages and demonstrated no cross-reactivity with multiple intestinal protozoa, including *Cryptosporidium parvum*, *Blastocystis hominis* and *Entamoeba* spp. Importantly, the assay identified several positive clinical samples that could not be confirmed by sequencing, suggesting that CRISPR-based detection may detect low-level infections that are not consistently confirmed by sequencing. The characteristics and diagnostic performance of the intestinal parasite CRISPR/Cas assays identified in this review are summarized in Table 2.

POC potential was assessed based on reported sample preparation requirements, equipment, workflow complexity, detection format, and extent of clinical or field validation. None of the reviewed assays had undergone validation under actual field conditions.

Across the studies reviewed, CRISPR-based assays demonstrated substantial variation in analytical performance, clinical validation, workflow complexity, and point-of-care applicability. Most platforms combined CRISPR detection with isothermal amplification, predominantly RPA or RAA, and generated results within approximately 60–90 min. Among the earlier studies, the Cryptosporidium parvum ReCTC assay demonstrated particularly strong potential for decentralized testing because it combined high analytical sensitivity with simplified DNA extraction and visual fluorescence or lateral-flow detection. Similarly, the *Giardia duodenalis*, *Clonorchis sinensis*, and *Fasciola hepatica* assays demonstrated high analytical sensitivity and practical visual readouts, although the extent of clinical validation varied among studies. In contrast, the *Schistosoma* SHERLOCK assay achieved exceptionally high analytical sensitivity and was evaluated using both stool and serum samples but involved a comparatively more complex workflow. More recently, the *Strongyloides stercoralis* RPA–CRISPR/Cas12a assay achieved a limit of detection of 500 fg/mL with high analytical specificity; however, it detected only three of six qPCR-positive stool samples, highlighting the need for further optimization and larger clinical validation studies.

The 2026 *C. sinensis* one-tube RPA–Cascade–Cas3 assay demonstrated greater operational integration, combining rapid sample preparation, closed-tube detection, visual readout, and an approximately 60-min sample-to-result workflow, with results highly concordant with PCR in 32 naturally infected fish samples. However, these were not human clinical specimens, and the assay primarily detected metacercaria in fish tissue, whereas diagnosis of human clonorchiasis typically relies on detection of parasite eggs or parasite-derived nucleic acids in stool. Therefore, despite its promising analytical and operational performance, its diagnostic performance in human clinical samples remains to be established.

## 5. Comparison of CRISPR/Cas Effector Systems

The choice of CRISPR effector protein is one of the most important factors influencing the performance of CRISPR-based diagnostic assays. Different Cas proteins possess distinct nucleic acid targets, collateral cleavage activities, reaction temperatures, and workflow requirements, all of which influence analytical sensitivity, assay simplicity, and suitability for point-of-care implementation. Among the currently available CRISPR systems, Cas12a has emerged as the predominant platform for intestinal parasite diagnosis, although recent studies have demonstrated the potential of Cas12b and Cas13a for specific applications. Understanding the strengths and limitations of each effector is therefore essential for selecting the most appropriate platform for future diagnostic development.

Among the CRISPR effectors currently available, Cas12a has emerged as the predominant platform for the molecular diagnosis of intestinal parasites. Following recognition of a target double-stranded DNA sequence adjacent to a protospacer adjacent motif (PAM), Cas12a undergoes conformational activation and exhibits indiscriminate collateral cleavage of nearby single-stranded DNA (ssDNA) reporter molecules. This collateral cleavage activity enables efficient signal amplification using fluorescent probes or lateral-flow strip (LFS) reporters, allowing highly sensitive nucleic acid detection without sophisticated instrumentation [16,17]. In addition, Cas12a operates efficiently at approximately 37 °C, closely matching the optimal temperature of recombinase polymerase amplification (RPA). This temperature compatibility allows amplification and CRISPR detection to be performed sequentially or integrated into simplified workflows, making Cas12a particularly suitable for point-of-care testing [5,18]. Furthermore, fluorescence- and LFS-based readouts permit rapid visual interpretation without requiring real-time PCR instrumentation, thereby reducing equipment requirements [20,24]. Consequently, Cas12a has become the most extensively validated CRISPR effector for parasite diagnosis and has been successfully applied to the detection of *Cryptosporidium parvum, Clonorchis sinensis, Fasciola hepatica*, and *Giardia duodenalis* [11,25,27,29,30]. Overall, Cas12a remains the most frequently applied CRISPR effector among the intestinal parasite diagnostic studies identified in this review., highlighting its current position as the leading platform for CRISPR-based intestinal parasite diagnostic.

Unlike Cas12a, Cas13a specifically recognizes RNA targets and, following target recognition, exhibits collateral cleavage of surrounding single-stranded RNA molecules. This property forms the basis of the SHERLOCK (Specific High-sensitivity Enzymatic Reporter Unlocking) platform, one of the earliest CRISPR-based diagnostic systems [20,33]. Cas13a-based assays are recognized for their exceptional analytical sensitivity and have demonstrated the ability to detect extremely low concentrations of nucleic acids, making them attractive for applications involving low parasite burdens or circulating parasite-derived nucleic acids [26]. In the only intestinal parasite study identified in this review, MacGregor et al. successfully applied the SHERLOCK platform for the diagnosis *of Schistosoma japonicum* and *S. mansoni* using both stool and serum samples, demonstrating excellent sensitivity and specificity. However, compared with Cas12a, Cas13a assays generally require additional processing steps, including in vitro transcription using T7 RNA polymerase to convert amplified DNA into RNA prior to CRISPR detection, resulting in a more complex workflow and the need for additional enzymes and reaction components [20,33]. These additional steps increase assay complexity, prolong processing time, and reduce suitability for decentralized testing. Therefore, despite its remarkable analytical sensitivity, Cas13a currently appears better suited for centralized laboratory diagnostics than routine point-of-care applications.

Although comparatively less explored, Cas12b represents an emerging alternative for molecular diagnostics. Like Cas12a, Cas12b recognizes double-stranded DNA targets and exhibits collateral cleavage of single-stranded DNA reporters. Some Cas12b orthologs function optimally at higher temperatures than Cas12a, which can complicate direct integration with conventional RPA/RAA conditions. Nevertheless, engineered reaction conditions and one-pot assay designs have enabled the integration of Cas12b with isothermal amplification, demonstrating its potential for simplified closed-tube diagnostic platforms [34]. This compatibility facilitates the development of one-pot assays, in which nucleic acid amplification and CRISPR detection occur simultaneously within a single closed reaction vessel. One-pot reactions minimize manual handling, reduce turnaround time, and substantially decrease the risk of carry-over contamination associated with opening reaction tubes after amplification [23,34]. Although only one intestinal parasite study has evaluated Cas12b for the detection of *Fasciola hepatica*, the successful integration of Cas12b with RAA demonstrates the feasibility of simplified closed-tube diagnostic platforms. As one-pot CRISPR systems continue to evolve, Cas12b may become an increasingly attractive candidate for next-generation sample-to-answer point-of-care diagnostics.

Unlike the single-effector Cas12 and Cas13 systems, Cas3 is a Class 1 type I CRISPR system that functions with the multisubunit Cascade complex, enabling sequence-specific target recognition followed by extensive DNA degradation [35]. Type I Cas3 systems have already demonstrated diagnostic potential; the Cas3-Operated Nucleic Acid detection (CONAN) platform enabled rapid detection of SARS-CoV-2 in clinical samples and discrimination of single-nucleotide variants, while a type I-A Cas3 platform was subsequently developed for HPV detection and demonstrated expanded PAM recognition and strong mismatch discrimination [12,36]. More recently, Bi et al. [32] developed a one-tube RPA–Cascade–Cas3 assay for *Clonorchis sinensis*, achieving a detection limit of 1 copy/µL within approximately 60 min while providing expanded PAM recognition and efficient degradation of RPA products, which may reduce carryover contamination.

## 6. Technical and Clinical Limitations

Despite the advances in CRISPR-based diagnostics for parasitic infections, several challenges remain before these technologies can be widely adopted in clinical and field settings. One of the principal concerns is the occurrence of false-negative results, which may arise from low parasite burdens, inefficient nucleic acid extraction, degradation or loss of target DNA, sequence variability within the target region, or inconsistent pre-amplification efficiency. Stool-derived DNA is particularly challenging because inhibitors and inefficient recovery of low-abundance parasite DNA can compromise downstream amplification [1,3,37]. The distinction between analytical sensitivity under controlled conditions and performance in clinical specimens is illustrated by the *Strongyloides stercoralis* RPA–CRISPR/Cas12a assay, which achieved an analytical limit of detection of 500 fg/mL but detected only three of six qPCR-positive stool samples [31]. This discrepancy may reflect low parasite burden, variable target abundance, stool-associated inhibitors, DNA extraction efficiency, or stochastic amplification at low target concentrations. In CRISPR-based assays, additional variability may arise during RPA/RAA and crRNA–target recognition. Target selection is therefore critical: conserved genomic regions with suitable protospacer adjacent motif (PAM) sequences can reduce the effects of sequence variation, while multicopy targets may improve sensitivity for low-intensity infections compared with single-copy targets because more target molecules are available for detection.

Importantly, no false-positive results were reported among the intestinal parasite CRISPR/Cas studies reviewed, whereas false-negative results were observed in some clinical evaluations. Nevertheless, the absence of reported false positives should be interpreted cautiously because most studies involved relatively small sample sizes and limited panels of non-target organisms. Potential false-positive signals may arise from nonspecific RPA/RAA, carryover contamination of amplified products, unintended crRNA recognition of closely related sequences, reporter instability, or ambiguous lateral-flow signals. The risk may be particularly relevant in highly sensitive assays incorporating pre-amplification, where contamination with small amounts of amplified DNA can generate detectable signals. These risks can be minimized through careful selection of parasite-specific and conserved target regions, optimization of primers and guide RNAs, testing against closely related organisms, inclusion of appropriate negative and no-template controls, and the use of closed-tube or one-pot workflows to reduce post-amplification contamination. Standardized interpretation criteria for fluorescence and lateral-flow readouts are also important to minimize subjective or nonspecific signal interpretation. Therefore, although false-positive results have not been a prominent finding in the studies reviewed, larger clinical studies incorporating broader specificity panels are required to establish diagnostic specificity under routine and field conditions.

However, it is important to note that these limitations are not unique to CRISPR-based diagnostics. Most false-negative results originate during the pre-analytical and analytical phases, particularly during sample collection, nucleic acid extraction, and amplification, which are common challenges shared by PCR, qPCR, LAMP, RPA, and other molecular diagnostic platforms as discussed previously [38,39]. Therefore, improvements in sample preparation and quality control are likely to have a greater impact on diagnostic accuracy than modifications to the CRISPR detection step alone.

Beyond these technical challenges, the current evidence base remains limited. Most published studies involve relatively small clinical cohorts and represent proof-of-concept investigations rather than large prospective clinical evaluations, limiting the generalizability of their findings. Moreover, multicentre validation across different endemic regions and genetically diverse parasite populations is lacking. Direct comparisons with established multiplex PCR assays are also scarce, making it difficult to determine the clinical advantages of CRISPR-based diagnostics over existing molecular methods. Furthermore, published studies have focused on only a limited number of parasites, primarily *Cryptosporidium parvum*, *Giardia duodenalis*, *Clonorchis sinensis*, *Fasciola hepatica*, and *Schistosoma* spp., while many clinically important intestinal protozoa and helminths remain unexplored. Finally, despite promising analytical performance, no CRISPR-based assay for intestinal parasite diagnosis has received regulatory approval or undergone formal WHO evaluation. Addressing these gaps through standardized protocols, large-scale multicentre studies, and independent clinical validation will be essential for translating CRISPR-based diagnostics into routine clinical and point-of-care practice.

Although CRISPR-based nucleic acid detection platforms demonstrate considerable promise as point-of-care (POC) diagnostic tools because of their high sensitivity, rapid turnaround time, and minimal equipment requirements, most current assays still require DNA extraction prior to analysis. This additional step increases assay complexity, prolongs processing time, and limits deployment in resource-constrained settings. None of the reviewed studies reported validation under actual field conditions, and laboratory-based DNA extraction or specialized equipment was still required. Therefore, these assays should be considered preliminary proof-of-concept platforms with potential for point-of-care application, rather than fully validated field-deployable diagnostic tests. Nevertheless, efforts to simplify sample preparation have already been reported. The CRISPR/Cas12a-based ReCTC assay developed for *Cryptosporidium parvum* utilized a rapid crude DNA preparation method based on N-lauroylsarcosine sodium salt (LSS) and a simple homemade DNA-binding column, reducing dependence on conventional commercial extraction kits while maintaining high diagnostic performance [11]. More recently, the *Clonorchis sinensis* RPA–Cascade–Cas3 assay incorporated rapid DAP-buffer-based sample preparation into a closed-tube workflow, further demonstrating progress toward simplified sample processing and integrated point-of-care-compatible detection. However, this approach has so far been evaluated in fish samples rather than human clinical specimens [32]. Together, these studies highlight the importance of simplifying nucleic acid preparation, while future development of extraction-free or fully integrated sample-to-answer platforms remains essential for translating CRISPR-based parasite diagnostics into routine clinical and field applications.

Another promising future direction is the detection of parasite-derived cell-free DNA (cfDNA) in serum or plasma. Several of the reviewed studies demonstrated high analytical sensitivity, suggesting that CRISPR/Cas systems may be suitable for detecting low concentrations of circulating parasite DNA. Such an approach could provide an alternative blood-based specimen for molecular detection and may facilitate disease monitoring; however, venipuncture remains invasive and should not be described as a non-invasive alternative to stool sampling. Future studies should evaluate the clinical performance of CRISPR-based circulating parasite DNA detection [26].

## 7. Point-of-Care Translation and Future Perspectives

According to the ASSURED and REASSURED criteria [40,41], current CRISPR-based assays for intestinal parasites demonstrate excellent analytical sensitivity and specificity, together with promising point-of-care characteristics, including isothermal amplification, portable readout formats, and minimal equipment requirements. However, most platforms remain limited by stool collection and processing, dependence on nucleic acid extraction, multistep workflows, assay durations of approximately 60–90 min, reagent stability, limited real-time connectivity, and insufficient large-scale field validation. Although assays targeting *Cryptosporidium parvum*, *Giardia duodenalis*, *Clonorchis sinensis*, *Fasciola hepatica*, and *Schistosoma* spp. exhibit considerable potential for decentralized diagnosis, none currently fulfils all ASSURED or REASSURED criteria. Future efforts should therefore focus on developing fully integrated sample-to-answer platforms that incorporate simplified or extraction-free sample preparation, one-pot amplification and detection, portable or smartphone-based readout systems, and robust clinical validation to enable routine implementation in resource-limited settings and decentralized healthcare facilities.

Lyophilization represents a promising strategy to improve reagent stability and reduce dependence on cold-chain transportation, and lyophilized CRISPR/Cas and isothermal amplification reagents have increasingly been explored for assay development. However, their translation into validated clinical diagnostic products remains limited. Integration of stabilized reagents with sample preparation, amplification, CRISPR detection, and readout within a closed disposable cartridge or cassette could ultimately enable GeneXpert-like sample-to-answer workflows for intestinal parasite diagnosis.

Multiplexing and amplification-free detection represent additional opportunities for advancing CRISPR-based diagnosis of intestinal parasites. Multiplex CRISPR platforms could enable the simultaneous detection and differentiation of multiple intestinal parasites within a single assay, which may be particularly valuable for stool specimens in settings where coinfections are common. Multiplex CRISPR detection has already been demonstrated in viral diagnostics, including the simultaneous discrimination of multiple viral targets, supporting the feasibility of adapting similar strategies to intestinal parasite panels [42]. In parallel, amplification-free CRISPR approaches could simplify workflows by eliminating the pre-amplification step, thereby reducing assay time, complexity, and the risk of amplicon contamination [21]. However, their application to intestinal parasites may be challenging because of the low abundance of parasite nucleic acids in clinical specimens, particularly in low-intensity infections. Therefore, improvements in CRISPR signal amplification and target enrichment may be required before amplification-free approaches can achieve sufficient sensitivity for routine intestinal parasite diagnosis.

## 8. Conclusions

**Figure 1:**
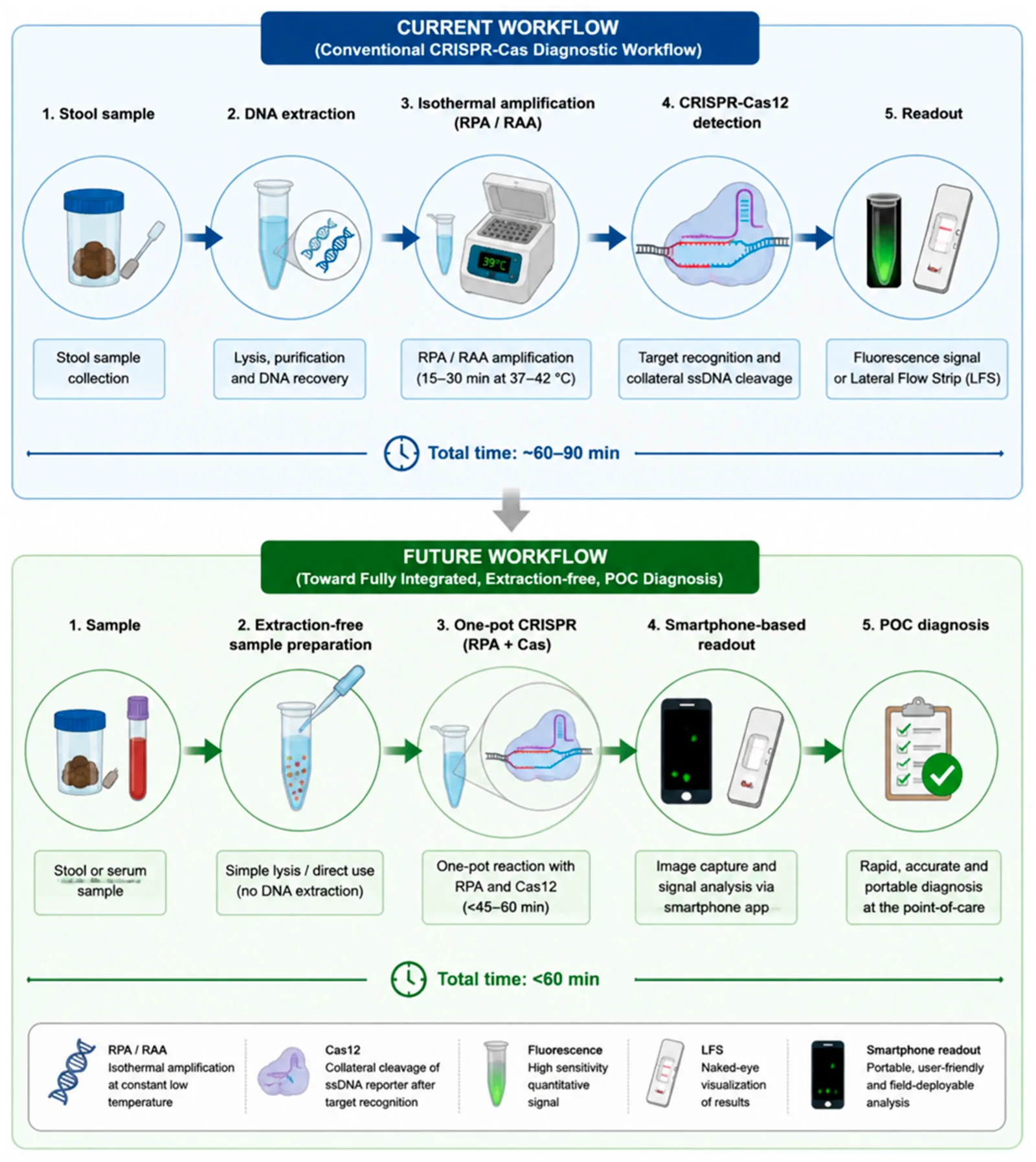
Current CRISPR/Cas diagnostic workflow and conceptual future sample-to-answer workflow for intestinal parasite detection.

Collectively, these advances highlight the considerable potential of CRISPR/Cas-based technologies to improve the diagnosis of intestinal parasitic infections, particularly in resource-limited and endemic settings. The integration of CRISPR/Cas systems with isothermal amplification, simplified nucleic acid extraction, and portable detection methods represents an important step toward rapid and accessible molecular diagnostics. However, despite promising analytical sensitivity and specificity, most existing assays remain at the proof-of-concept stage, with limited clinical validation and several technical and operational challenges. Future research should prioritize the development of fully integrated, extraction-free, sample-to-answer platforms incorporating stable reagents, simplified workflows, multiplex detection, and affordable readout systems. Large-scale, multicentre clinical validation and standardized comparisons with established diagnostic methods will be essential to demonstrate their reliability and clinical utility. Ultimately, addressing these limitations will facilitate the translation of CRISPR-based technologies into practical and field-deployable diagnostic tools for intestinal parasitic infections. (Figure 1).

## Figures and Tables

**Table 1:** Comparison of Conventional and Emerging Molecular Diagnostic Methods for Intestinal Parasites.

Method	Representative Analytical Detection Limit	Infrastructure Required	Approximate Turnaround Time	POC Potential	Reference
Microscopy	Parasite-dependent; generally expressed as cysts or eggs per gram of stool	Microscope, trained microscopist	30–60 min	Yes, with appropriate facilities	[6]
Antigen detection	Kit-dependent; generally expressed as antigen concentration or parasite equivalents	Minimal for rapid tests	15–30 min	Yes, for rapid-test formats	[7]
Conventional PCR	Target- and assay-dependent; report copies/reaction or parasite equivalents	Thermocycler, DNA extraction and electrophoresis equipment	2–4 h	Limited	[8]
qPCR	Example: approximately 8.8–30.1 DNA copies/µL for different intestinal parasite targets	Real-time PCR instrument and DNA extraction equipment	1.5–3 h	Limited	[9]
LAMP	Target-dependent; report copies/reaction or parasite equivalents	Heat block or portable heater; sample preparation	30–60 min	Potential	[10]
CRISPR/Cas	Examples: 1 oocyst/mL for C. parvum and target-dependent DNA-copy detection limits	Sample preparation, isothermal amplification equipment and fluorescence or lateral-flow detection	30–90 min, excluding some preparation steps	Potential; clinical field deployment remains unestablished	[11]

**Table 2:** Comparison of CRISPR/Cas-based diagnostic assays for the detection of intestinal and foodborne parasitic infections. The table summarizes target genes, Cas effectors, amplification strategies, DNA extraction methods, clinical validation, analytical sensitivity, specificity, diagnostic performance, readout modalities, assay duration, and point-of-care potential.

Parasite	Target Gene	CRISPR System	Amplification Method	DNA Extraction Method	Clinical Validation	Sensitivity (LOD)	Specificity	Diagnostic Performance	**Readout Format**	**Reported Assay Duration**	**POC Potential**	**Reference**
*Cryptosporidium parvum* (IId subtype family)	gp60	Cas12a	RPA (ReCTC assay)	Crude extraction using N-lauroy lsarcosine sodium salt (LSS) and homemade DNA-binding column	30 calf faecal samples + 8 human samples	1 copy target DNA; 1 oocyst/mL (fluorescence), 10 oocysts/mL (LFS)	No cross-reactivity with other *Cryptosporidium* subtype families or intestinal protozoa	100% agreement with nested PCR and sequencing	Fluorescence + LFS	~60 min	Potenial	[11]
*Clonorchis sinensis*	ITS1	Cas12a	RPA	TIANamp Genomic DNA Kit	50 human faecal + 50 fish samples	1 copy/µL DNA; 0.8 metacercaria/g fish (fluorescence)	No cross-reactivity with 7 pathogens	100% concordance with nested PCR	Fluorescence + Lateral Flow Strip (LFS)	~60 min	Potenial	[25]
*Schistosoma japonicum*/*S. mansoni*	Repetitive genomic targets	Cas13a (SHERLOCK)	RPA	QIAamp DNA Mini Kit, Maxwell LEV, Chemagic 360, E.Z.N.A. kits	Human and mouse stool/serum samples	0.2 pg/µL (*S. japonicum*); 0.001 pg/µL (*S. mansoni*)	Species-specific	Up to 100% sensitivity and specificity vs. qPCR	Fluorescence	~60 min	Partial	[26]
*Fasciola hepatica*	COX1	Cas12a	RPA	TIANamp Stool DNA Kit	143 sheep + 43 human faecal samples	0.122 copies/µL	No cross-reactivity with related parasites	98.39% agreement with qPCR/nested PCR	Fluorescence + LFS	~60–90 min	Potenial	[27]
*Fasciola hepatica*	COX1	Cas12b	RAA	TIANamp Stool DNA Kit	192 sheep faecal samples	2.6 copies/µL	No cross-reactivity	98.95% agreement with PCR/qPCR	Fluorescence + LFS	~60–90 min	Potenial	[28]
*Giardia duodenalis* Assemblages A & B (REPORT assay, 2024)	tpi	Cas12a (FnCas12a)	RPA (REPORT)	E.Z.N.A. Stool DNA Kit	60 human faecal samples	Assemblage A: 2.04 CFU/mL plasmid DNA; 10 trophozoites/g faeces. Assemblage B: 1.1 CFU/mL plasmid DNA; 10 cysts/g faeces	No cross-reactivity with assemblages C–F, *Cryptosporidium parvum*, *Enterocytozoon bieneusi*, *Blastocystis hominis*, and *Entamoeba* spp.	Detected all PCR-positive samples and additional sequencing-failed positives	Naked-eye fluorescence under blue light + LFS	~70 min	Potenial	[29]
*Giardia duodenalis*	Not specified	Cas12a	RPA	Faecal DNA extraction (not specified)	50 human + 70 cattle faecal samples	10^−1^ copies/µL	No cross-reactivity with 9 common pathogens	Positive rate: 2% (1/50 humans) and 47% (33/70 cattle); results consistent with nested PCR	Naked-eye fluorescence + LFS	~60 min	Potenial	[30]
*Strongyloides stercoralis*	Ss-NIE	Cas12a (LbaCas12a)	RPA	DNA extracted from human stool samples	6 qPCR-positive human stool samples	500 fg/mL gDNA	No cross-reactivity with *Ascaris lumbricoides*, *Trichuris trichiura*, *Wuchereria bancrofti*, *Loa loa*, or *Onchocerca volvulus*	3/6 qPCR-positive samples detected by RPA–CRISPR/Cas12a; pilot clinical validation	Fluorescence + visual blue-light readout	RPA 30 min + Cas12a 40–60 min	Potential; requires further clinical validation	[31]
*Clonorchis sinensis*	18S rRNA	Type I-A Cascade–Cas3 (TsiCas3)	RPA	Rapid DAP alkaline lysis	32 naturally infected fish samples; additionally tested in fish muscle spiked with 1–5 metacercaria	1 copy/µL; detection of a single spiked metacercaria	No signal with *Opisthorchis viverrini*, *Schistosoma japonicum*, *Fasciola hepatica*, or *Taenia solium*	Highly concordant with conventional PCR in 32 fish samples	Naked-eye fluorescence under blue/UV light + real-time fluorescence	~40 min amplification/detection; <60 min sample-to-result including rapid extraction	Potential; fish proof-of-concept	[32]

## Data Availability

No new data were created or analyzed during this study. Therefore, data sharing is not applicable to this article.

## Abbreviations

The following abbreviations are used in this manuscript:

**Abbreviation**	**Full Term**
ASSURED	Affordable, Sensitive, Specific, User-friendly, Rapid and Robust, Equipment-free, and Deliverable to End Users
Cas	CRISPR-associated protein
cfDNA	Cell-free DNA
crRNA	CRISPR RNA
CRISPR	Clustered Regularly Interspaced Short Palindromic Repeats
DETECTR	DNA Endonuclease-Targeted CRISPR Trans Reporter
DNA	Deoxyribonucleic Acid
LAMP	Loop-Mediated Isothermal Amplification
LFS	Lateral Flow Strip
LOD	Limit of Detection
LSS	*N*-Lauroylsarcosine Sodium Salt
PAM	Protospacer Adjacent Motif
PCR	Polymerase Chain Reaction
POC	Point-of-Care
POCT	Point-of-Care Testing
qPCR	Quantitative Polymerase Chain Reaction (Real-Time PCR)
RAA	Recombinase-Aided Amplification
REASSURED	Real-time Connectivity, Ease of Specimen Collection, Affordable, Sensitive, Specific, User-friendly, Rapid and Robust, Equipment-free, and Deliverable to End Users
REPORT	Recombinase Polymerase Amplification-Endonuclease CRISPR Trans Reporter
RPA	Recombinase Polymerase Amplification
SHERLOCK	Specific High-Sensitivity Enzymatic Reporter Unlocking
ssDNA	Single-Stranded DNA
